# RETRACTED: Alshabanah et al. Elastic Nanofibrous Membranes for Medical and Personal Protection Applications: Manufacturing, Anti-COVID-19, and Anti-Colistin Resistant Bacteria Evaluation. *Polymers* 2021, *13*, 3987

**DOI:** 10.3390/polym18131612

**Published:** 2026-06-29

**Authors:** Latifah Abdullah Alshabanah, Nada Omran, Bassma H. Elwakil, Moaaz T. Hamed, Salwa M. Abdallah, Laila A. Al-Mutabagani, Dong Wang, Qiongzhen Liu, Nader Shehata, Ahmed H. Hassanin, Mohamed Hagar

**Affiliations:** 1 Chemistry Department, College of Science, Princess Nourah Bint Abdulrahman University, Riyadh 11671, Saudi Arabia; laalsabanah@pnu.edu.sa (L.A.A.); laalmutbagani@pnu.edu.sa (L.A.A.-M.); 2 Science and Technology Institute, Wuhan Textile University, Wuhan 430073, China; nada.omranplus@gmail.com; 3 Centre of Smart Nanotechnology and Photonics (CSNP), SmartCI Research Centre, Alexandria University, Alexandria 21544, Egypt; nader83@vt.edu (N.S.); ahassanin2003@yahoo.com (A.H.H.); 4 Department of Medical Laboratory Technology, Faculty of Applied Health Sciences Technology, Pharos University in Alexandria, Alexandria 21321, Egypt; bassma.hassan@pua.edu.eg; 5 Industrial Microbiology and Applied Chemistry Program, Department of Botany & Microbiology, Faculty of Science, Alexandria University, Alexandria 21568, Egypt; moazt258@gmail.com; 6 Materials Science & Engineering Department, School of Innovative Design Engineering, Egypt-Japan University of Science and Technology (E-JUST), Alexandria 21934, Egypt; salwaabdallah17@gmail.com; 7 Key Laboratory of Textile Fiber and Products (Ministry of Education), Wuhan Textile University, Wuhan 430200, China; wangdon08@126.com; 8 Hubei Key Laboratory of Advanced Textile Materials & Application, Hubei International Scientific and Technological Cooperation Base of Intelligent Textile Materials & Application, Wuhan Textile University, Wuhan 430200, China; windlqz_2000@163.com; 9 Department of Engineering Mathematics and Physics, Faculty of Engineering, Alexandria University, Alexandria 21544, Egypt; 10 USTAR Bioinnovations Centre, Faculty of Science, Utah State University, Logan, UT 84341, USA; 11 Department of Physics, School of Engineering, Kuwait College of Science and Technology (KCST), Doha Superior Rd., Jahraa 13133, Kuwait; 12 Materials Science & Engineering Department, School of Innovative Design Engineering, Egypt-Japan University of Science and Technology (E-JUST), Alexandria 21934, Egypt; 13 Department of Textile Engineering, Faculty of Engineering, Alexandria University, Alexandria 21544, Egypt; 14 Chemistry Department, Faculty of Science, Alexandria University, Alexandria 21321, Egypt; 15 Chemistry Department, College of Sciences, Taibah University, Yanbu 30799, Saudi Arabia

The journal retracts the article titled, “Elastic Nanofibrous Membranes for Medical and Personal Protection Applications: Manufacturing, Anti-COVID-19, and Anti-Colistin Resistant Bacteria Evaluation” [1], cited above.

Following publication, concerns were brought to the attention of the Editorial Office regarding the integrity of a number of figures presented in this article [1].

In accordance with the journal’s standard procedures, an investigation was conducted by the Editorial Office and Editorial Board that identified duplications in Figures 1 and 6 and within Figure S6. Although the authors cooperated with the investigation, the explanations and supporting materials provided were not deemed sufficient by the Editorial Board to resolve the identified concerns. Consequently, the Editorial Board has lost confidence in the reliability of the reported findings and has decided to retract this publication, as per MDPI’s retraction policy.

This retraction was approved by the Editor-in-Chief of the journal *Polymers*.

The authors have been informed of this retraction and have indicated their disagreement with this decision.
